# Oxygen‐Driven Reconstruction Activates Quasi‐Single Pd Sites in Hollow PdAg Nanotubes for Zinc–Air and Fuel Cells

**DOI:** 10.1002/advs.202509329

**Published:** 2025-07-17

**Authors:** Zongge Li, Wenjie Tian, Kunsheng Hu, Yajie Guo, Xiaotan Tian, Wenjun Kang, Rui Li, Konggang Qu, Lei Wang, Fanpeng Meng, Huayang Zhang, Haibo Li

**Affiliations:** ^1^ Shandong Provincial Key Laboratory of Chemical Energy Storage and Novel Cell Technology School of Chemistry and Chemical Engineering Liaocheng University Liaocheng Shandong 252059 China; ^2^ School of Chemical Engineering The University of Adelaide Adelaide SA 5005 Australia

**Keywords:** electronic structure, fuel cell, oxygen reduction reaction, PdAg alloy, zinc‐air battery

## Abstract

Here, a template‐engaged galvanic replacement strategy is developed to construct hollow PdAg alloy nanotubes, where interfacial oxygen drives surface reconstruction and stabilizes quasi‐single Pd active sites. The interplay of atomic‐scale characterizations and theoretical calculations reveals that the oxygen‐induced atomic rearrangement downshifts the Pd *d*‐band center, optimizes the adsorption‐desorption energetics of ORR intermediates, and lowers the energy barrier for ^*^OH desorption. The optimized Pd_0.30_@Ag catalyst achieves an onset potential of 0.951 V and a half‐wave potential of 0.868 V in alkaline media, surpassing commercial Pt/C even at an ultra‐low Pd loading (3 wt.%). Furthermore, Pd_0.30_@Ag‐based electrodes deliver outstanding performance in both zinc‐air batteries (ZABs) and anion‐exchange membrane fuel cells (AEMFCs), demonstrating high power densities, excellent cycling stability, and strong potential for scalable platinum‐free energy conversion devices. This work provides a general strategy for engineering interface‐confined active sites through surface reconstruction, offering new insights into the rational design of next‐generation electrocatalysts.

## Graphical abstract

A galvanic replacement strategy is employed to fabricate hollow PdAg alloy nanotubes, where interfacial oxygen triggers surface reconstruction and stabilizes quasi‐single Pd active sites. This atomic‐level engineering optimizes electronic configurations and enriches exposed catalytic centers, significantly boosting oxygen reduction reaction activity.

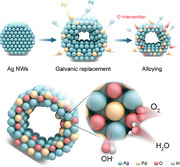

## 1 Introduction

Developing highly active and stable electrocatalysts to overcome the sluggish kinetics of the cathodic oxygen reduction reaction (ORR) is critical for enhancing the performance of metal‐air batteries and fuel cells.^[^ ^1^, ^2^, ^3^ ^]^ The slow reaction kinetics primarily result from the multi‐step, four‐electron transfer process involved in O_2_ reduction, which is strongly influenced by the adsorption and desorption behavior of intermediates on the catalyst surface, as described by the Sabatier principle.^[^ ^4^, ^5^ ^]^ Currently, platinum (Pt)‐based catalysts are considered the benchmark for ORR due to their optimal binding strength with oxygen‐containing intermediates.^[^ ^6^, ^7^, ^8^ ^]^ However, the widespread application of Pt‐based catalysts is limited by their high cost, low abundance, and insufficient long‐term durability.^[^ ^9^, ^10^, ^11^, ^12^, ^13^ ^]^ To address these challenges, the development of Pt alternatives that are more abundant and industrially scalable has gained considerable attention.^[^ ^14^ ^]^ Among the potential candidates, palladium (Pd) has emerged as a promising option due to its similar crystal and electronic structures and comparable chemical reactivity to Pt, making it a viable substitute for ORR electrocatalysis.^[^ ^15^, ^16^ ^]^

The significant affinity of Pd for oxygen‐containing intermediates often results in sluggish conversion and desorption of these species on Pd‐based electrocatalysts, requiring more negative potentials and leading to inferior ORR performance compared to Pt‐based catalysts, as widely reported.^[^ ^17^, ^18^, ^19^ ^]^ Therefore, it is crucial to design Pd‐based catalysts with highly efficient active sites. In this context, Ag has emerged as a strategic alloying element due to its dual role in structural templating and electronic modulation. Ag‐based bimetallics have demonstrated enhanced ORR activity by tuning the electronic structure of the active metal. Specifically, Ag‐Pd nano‐configurations have shown promising performance due to *d*‐band center shifts predicted by physics‐based modeling, which optimize the adsorption energy of oxygen intermediates.^[^ ^20^ ^]^ In addition, precious metal catalysts are susceptible to poisoning by reaction intermediates generated during the ORR, causing a decline in both activity and stability.^[^ ^21^ ^]^ To address these challenges, significant efforts have been made to develop palladium‐based multi‐component alloys with various morphologies, including sphere, core–shell structures, and nanotubes. These designs aim to reduce noble metal usage, improve the dispersion of active sites, and enhance catalytic performance.^[^ ^22^, ^23^, ^24^, ^25^, ^26^ ^]^ Remarkably, 1D nanowires (NWs) offer several advantages, such as superior electronic conductivity, high resistance to Ostwald ripening, and the ability to prevent the aggregation and dissolution of Pd‐based alloy nanoparticles, making them promising for electrocatalytic applications.^[^ ^27^ ^]^ The construction of core–shell with hollow structures can increase the accessible active surface area at the three‐phase interface, thereby improving the reaction kinetics.^[^ ^28^ ^]^ However, the synthesis of 1D Pd‐based NWs with hollow structures remains challenging due to their high surface energy and limited thermodynamic stability. Furthermore, the influence of high surface energy on oxygen species adsorption, which could significantly impact ORR performance, is often overlooked in previous studies.

Here, we developed a template replacement strategy to successfully synthesize hollow PdAg alloy NW catalysts, denoted as Pd_x_Ag. The resulting catalysts feature a high fraction of exposed PdAg (111) crystal facets, which are stabilized by surface‐adsorbed oxygen atoms. This surface modification not only reduces the high surface energy but also creates abundant Pd‐based active sites with enhanced selectivity, activity, and stability for the ORR. The presence of oxygen atoms on the PdAg alloy surface was confirmed by the EDS mapping, XPS, and XAFS analyses. The DFT analysis indicated that oxygen adsorption at the PdAg alloy interface significantly alters the charge distribution and electronic structure of the active sites, particularly by tuning the *d*‐orbital electrons of the Pd atoms. As a result, the *d*‐band center is negatively shifted, thereby weakening the adsorption strength of ^*^OH intermediate and improving ORR kinetics. As a result, Pd_0.30_@Ag exhibits ORR activity comparable to commercial Pt/C (20 wt.%), achieving an onset potential of 0.951 and a half‐wave potential of 0.868 V versus RHE. When composited with carbon to reduce the noble metal loading, the catalyst maintains stable performance even with a metal mass fraction as low as 3%. These findings suggest that Pd_0.30_@Ag is a promising candidate for replacing Pt in ORR applications and holds great potential for scale‐up applications.

## 2 Results and Discussion

### 2.1 Preparation and Characterization of Pdx@Ag

**Figure 1:**
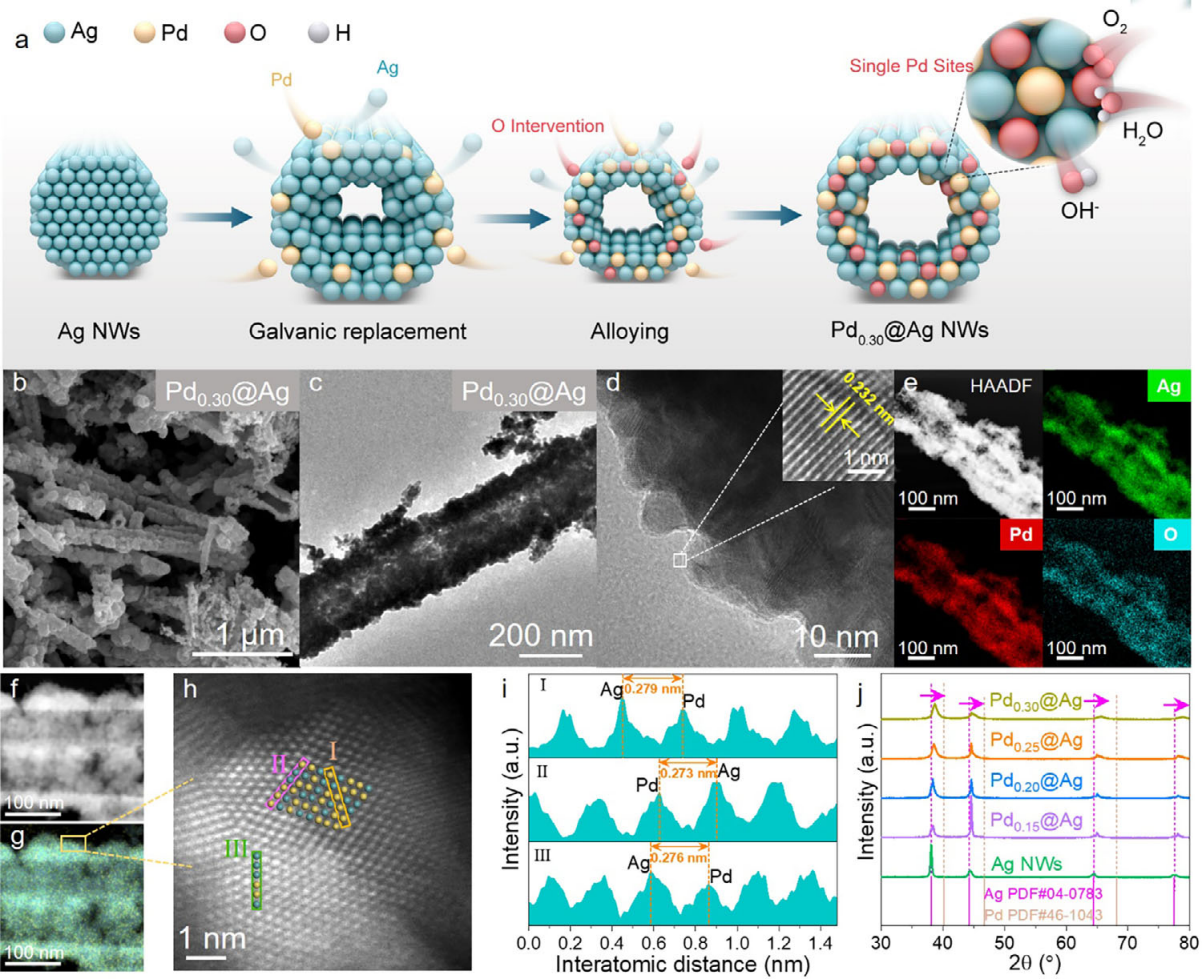
Morphology and crystalline characterizations. a) Schematic representation of the synthesis of the PdAg Alloyed NWs; b) SEM, c) TEM, d) HRTEM, and e) EDS mapping of Pd_0.30_@Ag; f) HAADF‐STEM with g) elemental mapping and h) aberration‐corrected STEM and i) interatomic distance images of Pd_0.30_@Ag; j) XRD patterns of Pd_x_@Ag.

The synthesis process of Pd_x_@Ag is shown schematically in **Figure** **1**a and involves two main steps: the preparation of Ag nanowires (Ag NWs) and the subsequent Pd displacement reaction. The galvanic replacement proceeds at room temperature and atmospheric pressure, with Pd^2+^ ions being reduced and deposited onto the Ag nanowire surface while Ag atoms are oxidized and released into the solution. In this process, the Ag nanowires act as a sacrificial template, guiding the deposition and shaping of the final PdAg alloy structure. Notably, as the molar ratio of Pd^2+^ increases, the solution color transitions until, at a ratio of 0.30, the solution ceases to become clearer, indicating the saturation of the galvanic replacement and the establishment of equilibrium where excessive Pd salt could not be reduced (Figure S1, Supporting Information). The morphology of the samples was observed using scanning electron microscopy (SEM) and transmission electron microscopy (TEM). As shown in Figure 1b and Figure S2 (Supporting Information), Pd_x_@Ag exhibits a 1D hollow nanotube structure with a rough surface, which is favorable for exposing more catalytically active sites. TEM images of samples with different feed ratios (Figure 1c; Figure S3, Supporting Information) clearly show the morphological evolution during the displacement reaction. The initially smooth and uniform Ag NWs gradually developed roughened surfaces and hollow interiors as the Pd content increased. The stabilization of the crystalline phase and morphology observed by TEM also confirms that the galvanic replacement is complete.

High‐resolution TEM (HRTEM) image (Figure 1d) revealed a lattice spacing of 0.232 nm for Pd_0.30_@Ag, which falls between the (111) interplanar distances of fcc‐Ag (0.237 nm) and fcc‐Pd (0.225 nm), further confirming the alloy formation.^[^ ^29^ ^]^ Elemental mapping using an energy‐dispersive spectrometer (EDS) for Pd_0.30_@Ag in Figure 1e–g reveals a uniform distribution of Pd and Ag throughout the hollow nanotubes with an average diameter of ≈200 nm, verifying the PdAg alloying feature. Notably, a significant amount of oxygen species was observed on the surface of the alloy, indicating surface oxidation during synthesis. Atomic‐scale characterization of the PdAg alloy was performed using aberration‐corrected high‐angle annular dark‐field scanning transmission electron microscopy (AC‐HAADF‐STEM). The AC‐HAADF‐STEM image (Figure 1h) clearly resolves individual Pd (Z = 46) and Ag (Z = 47) atoms, where the brighter contrast corresponds to Ag atoms due to their higher atomic number, confirming the atomic‐level alloying (Figure S4, Supporting Information). The measured nearest‐neighbor atomic spacing of 2.76 ± 0.03 Å corresponds to the (111) interplanar distance of a relaxed PdAg solid solution (Figure 1i), as verified by geometric phase analysis in Figure S5 (Supporting Information).

The crystalline phases of Pd_x_@Ag with varying Pd‐to‐Ag feed ratios were identified by powder X‐ray diffraction (XRD) pattern (Figure 1j). The diffraction peaks of Pd_x_@Ag are located between those of face‐centered cubic (fcc) Ag (PDF#04‐0783) and fcc Pd (PDF#46‐1043), confirming the formation of PdAg alloys with an fcc structures.^[^ ^30^ ^]^ Furthermore, a slight positive shift in the diffraction peak with increasing Pd content indicates enhanced alloying between Pd and Ag.

### 2.2 Surface Composition and Fine Structure of Catalysts

**Figure 2:**
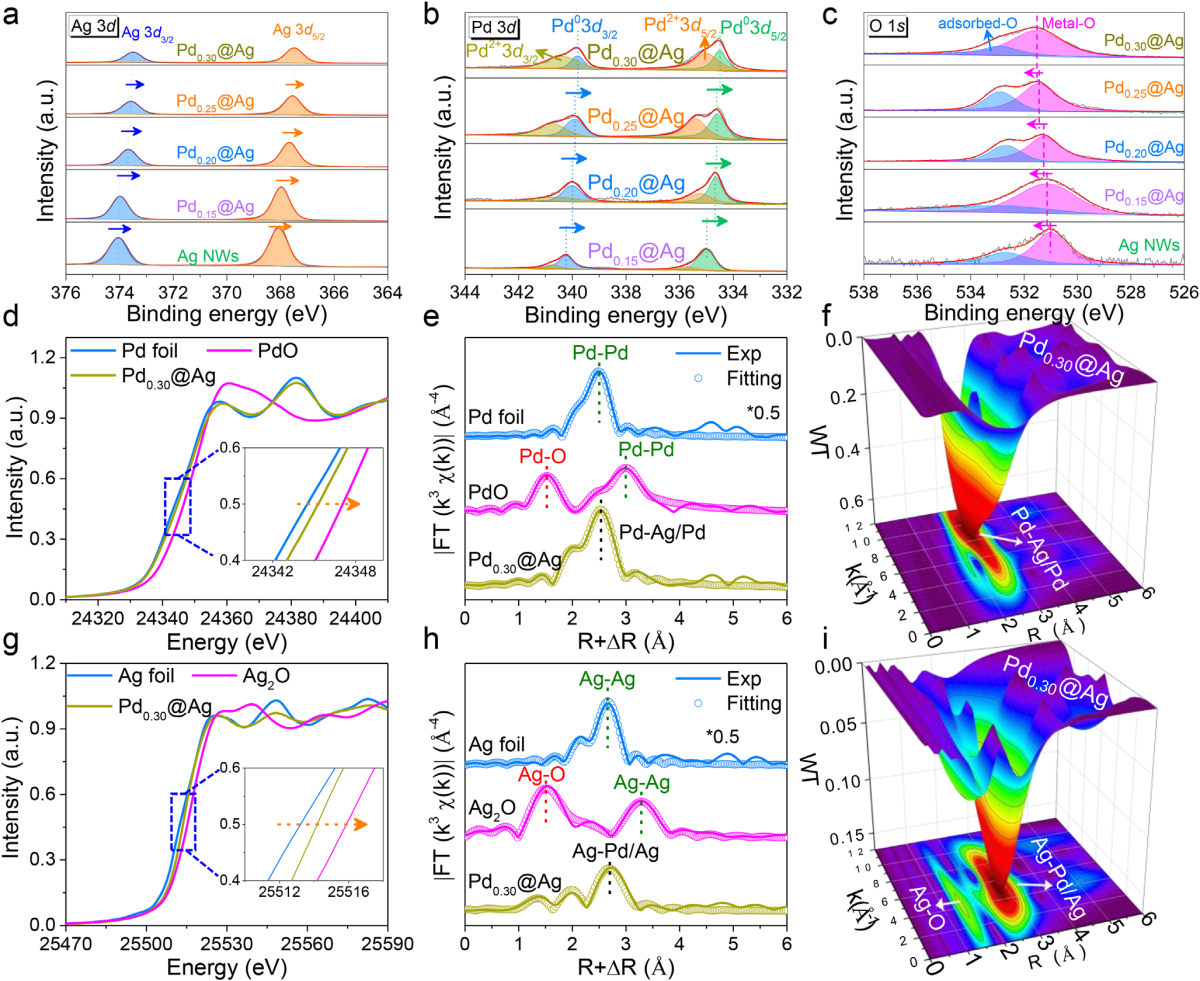
Surface composition and structure characterization of Pd_x_@Ag: a) Ag 3*d*, b) Pd 3*d*, and c) O 1*s* XPS spectra of Pd_x_@Ag and Ag NWs. d) XANES spectra at the Pd K‐edge of Pd_0.30_@Ag and reference samples (including Pd foil and PdO); e) the FT‐EXAFS data for three Pd‐relevant samples with their fitting curves; f) WT‐EXAFS image of Pd K‐edge for Pd_0.30_@Ag. g) XANES spectra at the Ag K‐edge of Pd_0.30_@Ag and reference samples (including Ag foil and Ag_2_O); h) the FT‐EXAFS data for three Ag‐relevant samples with their fitting curves; i) WT‐EXAFS image of Ag K‐edge for Pd_0.30_@Ag.

The surface chemical composition and valence state of the catalysts were analyzed by X‐ray photoelectron spectroscopy (XPS). The XPS survey of Pd_x_@Ag revealed the presence of Pd, Ag, O, and trace amounts of C (Figure S6a, Supporting Information), where the carbon signal mainly originated from the reduction of acetyl groups present in the precursors. Inductively coupled plasma optical emission spectrometry (ICP‐OES) confirmed that the Pd mass fraction in Pd_0.15_@Ag, Pd_0.20_@Ag, Pd_0.25_@Ag, and Pd_0.30_@Ag were 19.18%, 23.99%, 25.47%, and 32.01%, respectively (Figure S6b, Supporting Information), reflecting a more thorough progression of the displacement reaction with higher Pd feedstock. The high‐resolution Ag 3*d* spectrum in **Figure** **2**a displays two characteristic peaks at approximately 367.9 and 374.1 eV, corresponding to Ag 3*d*_5/2_ and Ag 3*d*_3/2_, respectively.^[^ ^31^ ^]^ The Pd 3*d* spectra of the Pd_x_@Ag (Figure 2b) can be deconvoluted into two doublets: Pd^0^ 3*d*_5/2_, Pd^0^ 3*d*_3/2_ states and Pd^2+^ 3*d*_5/2_ and Pd^2+^ 3*d*_3/2_ states.^[^ ^32^ ^]^ In the high‐resolution O 1*s* spectra (Figure 2c), two distinct peaks are observed corresponding to metal─O bonds (∼530.9 eV) and adsorbed‐O (∼532.6 eV).^[^ ^33^, ^34^ ^]^ The binding energies of both Ag and Pd shifted to lower values with increasing Pd content, suggesting a decrease in the surface valence states of both metals. In contrast, the metal─O binding energy follows the opposite trend, increasing with higher Pd incorporation. This indicates that Pd doping modulates the electron transfer between the interfacial metals and oxygen species, leading to electronic structure changes that could influence catalytic performance.

X‐ray absorption near‐edge structures (XANES) and extended X‐ray absorption fine spectroscopy (EXAFS) were used to investigate the average oxidation state and local coordination environment of the PdAg alloy. Several reference samples were also measured for comparison, including Pd and Ag metal foils and their corresponding oxides (PdO, Ag_2_O). As shown in Figure 2d, the Pd K‐edge absorption threshold of Pd_0.30_@Ag lies between those of Pd foil and PdO, suggesting that Pd in Pd_0.30_@Ag exhibits a higher average oxidation state than metallic Pd. Figure 2e shows the Fourier‐transformed EXAFS (FT‐EXAFS) spectra of Pd in Pd_0.30_@Ag and the reference samples. The major peak of Pd foil detected in R‐space is concentrated at ∼2.5 Å, which corresponds to the Pd─Pd bond of the first coordination shell.^[^ ^35^ ^]^ The PdO shows a strong peak at ∼1.6 Å, which corresponds to the Pd─O bond and a second‐shell Pd─Pd bond at ∼3.9 Å. The wavelet transforms EXAFS (WT‐EXAFS) analysis further confirmed these observations (Figure 2f; Figure S7a,b, Supporting Information), where the intensity maximum for Pd in Pd_0.30_@Ag appears at ≈8.0 Å^−1^ in k‐space and ≈2.5 Å in R‐space, attributable to Pd─Pd/Ag bonding.^[^ ^36^ ^]^

The Ag K‐edge XANES spectrum shows that the absorption threshold of Pd_0.30_@Ag falls between those of Ag foil and Ag_2_O (Figure 2g), indicating that Ag in Pd_0.30_@Ag carries partial positive charges, consistent with the XPS results for surface oxygen species. The FT‐EXAFS spectra of Ag atoms showed that Ag foil exhibits a strong Ag─Ag bond at ≈2.7 Å, while Ag_2_O shows a characteristic Ag─O bond at ≈1.5 Å (Figure 2h). In Pd_0.30_@Ag, the observed peak at ∼2.5 Å is attributed to Ag─Pd/Ag bonding. The WT‐EXAFS contour plot of Pd_0.30_@Ag displays an intensity maximum at ≈4.0 Å^−1^ in k‐space, corresponding to Ag─O scattering (Figure 2i), whereas Ag foil and Ag_2_O standards (Figure S7c,d, Supporting Information) show a stronger intensity at ≈6.0 Å^−1^ due to metallic Ag─Pd/Ag scattering.^[^ ^37^ ^]^ Notably, the similarity in peak position at ∼4 Å for both Ag─O and Ag─Ag features in the k‐space of Ag_2_O aligns with the physical scenarios where surface oxygen strongly interacts with silver, particularly in oxidized or highly oxygen‐affinitive environments (Figure S7d, Supporting Information).^[^ ^38^ ^]^ The strong affinity of surface oxygen atoms induces peak overlap or similarity in the WT analysis, indicating that the surface oxygen in PdAg alloys is predominantly introduced by Ag.

The EXAFS fitting results (Table S1, Supporting Information) reveal that the coordination environment of Pd consists of Pd─O, Pd─Pd, and Pd─Ag bonds with coordination numbers of 1.1 ± 0.2, 7.1 ± 0.4, and 4.1 ± 0.3, respectively. The Ag coordination environment includes Ag─O, Ag─Ag, and Ag─Pd bonds, indicating the presence of oxygen species at the PdAg alloy interface.

### 2.3 Electrochemical Characterization in Alkaline Electrolyte

**Figure 3:**
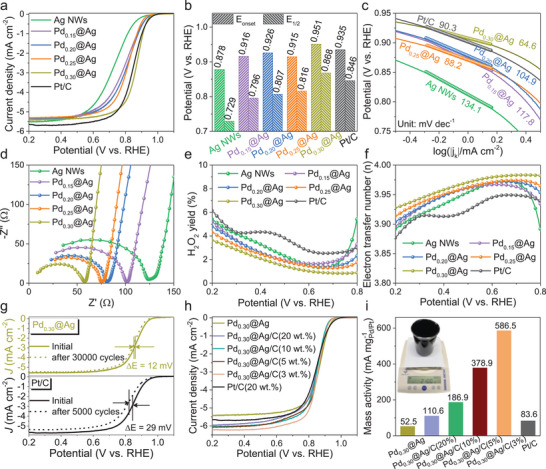
Oxygen electrochemical reduction measurements of Ag NWs, Pd_x_@Ag, and Pt/C. a) ORR LSV curves without *iR* correction, b) histograms of comparative E_onset_ and E_1/2_, and c) Tafel plots of Ag NWs, Pd_x_@Ag, and Pt/C. d) EIS Nyquist plots, e) H_2_O_2_ yield, and f) electron transfer number calculated by RRDE. g) Long‐term cycling stability via the ADT method of Pd_0.30_@Ag and Pt/C. h) ORR LSV curves of Pd_0.30_@Ag under carbon loading conditions for mass fractions of Pd for 20%, 10%, 5%, 3% and Pt/C (20 wt.%) and i) their mass activity.

The ORR performance of all catalysts was first evaluated by cyclic voltammetry (CV) with a scan rate of 20 mV s^−1^ (Figure S8, Supporting Information). Clear ORR reduction peaks were observed under O_2_‐saturated conditions, while negligible activity appeared in N_2_‐saturated electrolyte. Among the catalysts, Pd_0.30_@Ag exhibited the most positive peak (∼0.82 V vs RHE), suggesting excellent ORR catalytic activity. Linear sweep voltammetry (LSV) was further performed using a rotating disk electrode (RDE) at 1600 rpm (**Figure** **3**a). The pure Ag NWs exhibited the most sluggish ORR activity with an onset potential (E_onset_) of ≈0.82 V and a half‐wave potential (E_1/2_) of ≈0.73 V. However, the formation of PdAg alloys significantly improved the ORR performance. As shown in Figure 3b, the Pd_0.30_@Ag exhibits an excellent Eonset of 0.951 V and E_1/2_ of 0.868 V, which outperforms the Pd_0.25_@Ag (E_onset_ = 0.915 V, E_1/2_ = 0.816 V), Pd_0.20_@Ag (E_onset_ = 0.926 V, E_1/2_ = 0.807 V), Pd_0.15_@Ag (E_onset_ = 0.916 V, E_1/2_ = 0.796 V), and commercial Pt/C (E_onset_ = 0.935 V, E_1/2_ = 0.846 V). The Tafel slope of Pd_0.30_@Ag (≈64.6 mV dec^−1^) was lower than that of Pd_0.25_@Ag (≈88.2 mV dec^−1^), Pd_0.20_@Ag (≈104.9 mV dec^−1^), Pd_0.15_@Ag (≈117.8 mV dec^−1^), Ag NWs (≈134.1 mV dec^−1^) and Pt/C (≈90.3 mV dec^−1^) (Figure 3c), indicating that Pd_0.30_@Ag possesses the optimal ORR kinetics.^[^ ^39^ ^]^

Electrochemical impedance spectroscopy (EIS) measurement further confirmed the superior charge transfer properties of Pd_0.30_@Ag, as evidenced by its smallest arc radius in the Nyquist plot (Figure 3d). The equivalent circuit fitting (Figure S9, Supporting Information) revealed that Pd_0.30_@Ag exhibited the lowest charge transfer resistances (R_ct_ = 49.2 Ω cm^−2^) compared to Pd_0.25_@Ag (62.4 Ω cm^−2^), Pd_0.20_@Ag (69.5 Ω cm^−2^), Pd_0.15_@Ag (90.8 Ω cm^−2^) and Ag NWs (109.8 Ω cm^−2^) (Table S2, Supporting Information), further validating its superior charge transfer capability during the ORR.^[^ ^40^ ^]^

To further investigate the catalytic mechanism of PdAg alloy catalysts, LSV curves were conducted at rotation speeds ranging from 400 to 1600 rpm (Figure S10a–f, Supporting Information). The E_onset_ remains constant as the rotation speed increases, while the current density increases proportionally, confirming that the ORR follows a diffusion‐controlled process. The Koutecky–Levich (K–L) curves of all catalysts exhibited good linearity and parallel trends across different potentials, with a calculated electron transfer number (n) of ≈4.0 (Figure S10g–l, Supporting Information), suggesting that each oxygen molecule follows the standard 4‐electron transfer process. The rotating ring disk electrode (RRDE) tests further demonstrated that the Pd_x_@Ag catalysts generate a low H_2_O_2_ yield (< 6.0%) over a wide potential range of 0.2–0.8 V (Figure 3e; Figure S11, Supporting Information), with calculated n values ranging from 3.90 to 4.00 (Figure 3f), consistent with the K–L analysis. The electrochemical stability of Pd_0.30_@Ag and Pt/C was evaluated by accelerated durability testing (ADT). After 30 000 cycles, Pd_0.30_@Ag displayed a slight negative shift of ≈12 mV in its half‐wave potential (Figure 3g), significantly smaller than the ≈29 mV shift observed for Pt/C after 5000 cycles, indicating excellent long‐term stability.

### 2.4 Prospects for Industrial Applications

To assess the practical applicability of Pd_0.30_@Ag, the catalyst was mechanically mixed with conductive carbon black (XC‐72) to prepare carbon‐supported PdAg alloy catalysts. These composites were designated as Pd_0.30_@Ag/C(X), where X represents the Pd mass fraction (20%, 10%, 5%, or 3 wt.%) determined by ICP analysis. The LSV results showed that the ORR performance of these carbon‐supported catalysts remained comparable to the carbon‐free Pd_0.30_@Ag catalyst (Figure 3h). This demonstrates that combining Pd_0.30_@Ag with XC‐72 enables cost‐effective large‐scale catalyst production without compromising performance.

Pd_0.30_@Ag/C catalyst with the lowest Pd content (3 wt.%) achieved the highest mass activity of 586.5 ${\mathrm{mA}\;\mathrm{Pd}}_{\mathrm{mg}}^{-1}$ at 0.85 V (Figure 3i), which is 7 times that of commercial Pt/C (83.6 ${\mathrm{mA}\;\mathrm{Pt}}_{\mathrm{mg}}^{-1}$). Moreover, all Pd_0.30_@Ag/C(X) series catalysts can be synthesized on a hundred‐gram scale (inset of Figure 3i), highlighting their scalability and commercial potential.

### 2.5 Theoretical Evidence for the Surface Reconstruction of Oxygen‐Confined Quasi‐Single Pd Sites

**Figure 4:**
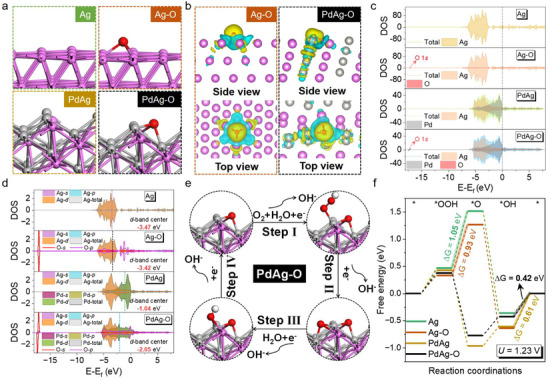
Theoretical investigation of ORR mechanisms based on catalytic model sites. a) Structural diagrams of the molecular models of Ag (111), Ag‐O (111), PdAg (111), and PdAg‐O (111). b) Differential charge analysis of Ag‐O (111) and PdAg‐O (111). c) Total density of states (TDOS) and contributions of constituent elements. d) DOS for the *d* orbital with *d*‐band centers of the atomic active site. e) Schematic diagrams of the ORR pathways of PdAg‐O (111). f) Calculated adsorption energies for the ORR intermediates.

To gain deeper insights into the positive effects of interface O modification on the ORR of PdAg alloys, first‐principles calculations were carried out using the VASP program package based on density functional theory (DFT). A model structure representative of the PdAg alloy phase was constructed for theoretical simulations. Specifically, four models were employed: Ag (111), Ag‐O (111), PdAg (111), and PdAg‐O (111) (**Figure** **4**a). The well‐established 4‐electron ORR pathway proposed by Nørskov was adopted as follows:^[^ ^41^ ^]^

(1) $$*O_{2}+H_{2}O\left(l\right)+e^{-}\to *\mathrm{OOH}+OH^{-}$$

(2) $$*\mathrm{OOH}+e^{-}\to *O+OH^{-}$$

(3) $$*O+H_{2}O\left(l\right)+e^{-}\to *\mathrm{OH}+OH^{-}$$

(4) $$*\mathrm{OH}+e^{-}\to OH^{-}+*$$

where * represents the active sites on the catalyst surface, ^*^OH, ^*^O, and ^*^OOH correspond to the adsorbed reaction intermediate during the ORR. The Gibbs free energy change (ΔG) for each reaction step under the applied external electrode potential (*U*) can be calculated using Equations. (5).

(5) ΔG=ΔE+ΔZPE−TΔS−neU

where ∆E, ∆ZPE, ∆S, and neU represent the total energy, the zero‐point vibrational energy, the entropy, and the contribution of electrons transfer in the electrode with electrode potential to ΔG, respectively, and T was adopted as 298.15 K. The theoretical overpotential at the equilibrium potential was determined according to Equations. (6).

(6) $$\eta =1.23-\left|\Delta G_{\max}/e^{-}\right|$$

where ΔG_max_ was the maximum free energy change of adjacent electronic steps.

To analyse the charge distribution and quantify the number of electron transfers, differential charge analysis and Bader charge calculations were performed. Bader charge analysis indicates that the adsorbed O atom on the Ag (111) surface forms bonds with three Ag atoms, with each Ag atom transferring 0.20 e^−^ to the O atom (Figure S12a, Supporting Information). This results in a relatively uniform charge distribution, as reflected in the differential charge density (Figure 4b). In contrast, on the PdAg alloy surface, the interfacial O atom is coordinated with two Pd atoms and one Ag atom. The Pd atoms transfer 0.28 e^−^ and 0.24 e^−^, respectively, while the Ag atom contributes 0.16 e^−^ (Figure S12b, Supporting Information). This leads to an asymmetric charge distribution, as shown in Figure 4b, indicating that interfacial oxygen induces localized and directional electron transfer, thereby altering the surface electronic structure.

The projected density of states (PDOS) and total density of states (TDOS) analyses for the four models revealed substantial electron density near the Fermi level, mainly contributed by the metallic components, suggesting the availability of mobile charge carriers for facilitating the reaction (Figure 4c). These results highlight the key role of metal atoms in enabling efficient proton‐coupled electron transfer during the ORR.^[^ ^42^ ^]^

The electronic structure of the active sites directly influences catalytic performance by modulating orbital contributions. As shown in Figure 4d, for Ag (111) and Ag‐O (111), the TDOS is dominated by *d*‐orbital electrons, and oxygen adsorption causes minimal changes to the *d*‐band center. In contrast, the PdAg (111) and PdAg‐O (111) systems exhibit significant changes in the electronic structure due to the combined contributions of Pd and Ag *d*‐orbitals. Upon O adsorption, the *d*‐band becomes broader and shifts negatively from −1.04 to −2.05 eV. This downshift reduces the adsorption strength of ORR intermediates, facilitating their desorption and accelerating the reaction kinetics.^[^ ^43^ ^]^

Following the typical 4‐electron ORR pathway, involving OOH^*^, O^*^, and OH^*^ intermediates (Figure 4e; Figure S13, Supporting Information), Gibbs free energy change (ΔG) diagrams were constructed under an applied potential of 1.23 V for Ag (111), Ag‐O (111), PdAg (111) and PdAg‐O (111) models (Figure 4f). For the Ag‐based surfaces, the rate‐determining step (RDS) is the conversion of ^*^OOH to ^*^O. In contrast, for Pd‐based systems, the RDS is the desorption of ^*^OH. Importantly, PdAg‐O exhibited the lower overpotential (ƞ = 0.42 eV) compared to PdAg (ƞ = 0.61 eV), Ag (ƞ = 1.05 eV), and Ag‐O (ƞ = 0.93 eV). These results confirm that interfacial oxygen not only modulates the electronic structure but also enhances the intrinsic activity of Pd sites, significantly improving the ORR electrocatalytic performance.

### 2.6 Electrochemical Performance in Zinc‐Air Batteries and Anion‐Exchange Membrane Fuel Cells

**Figure 5:**
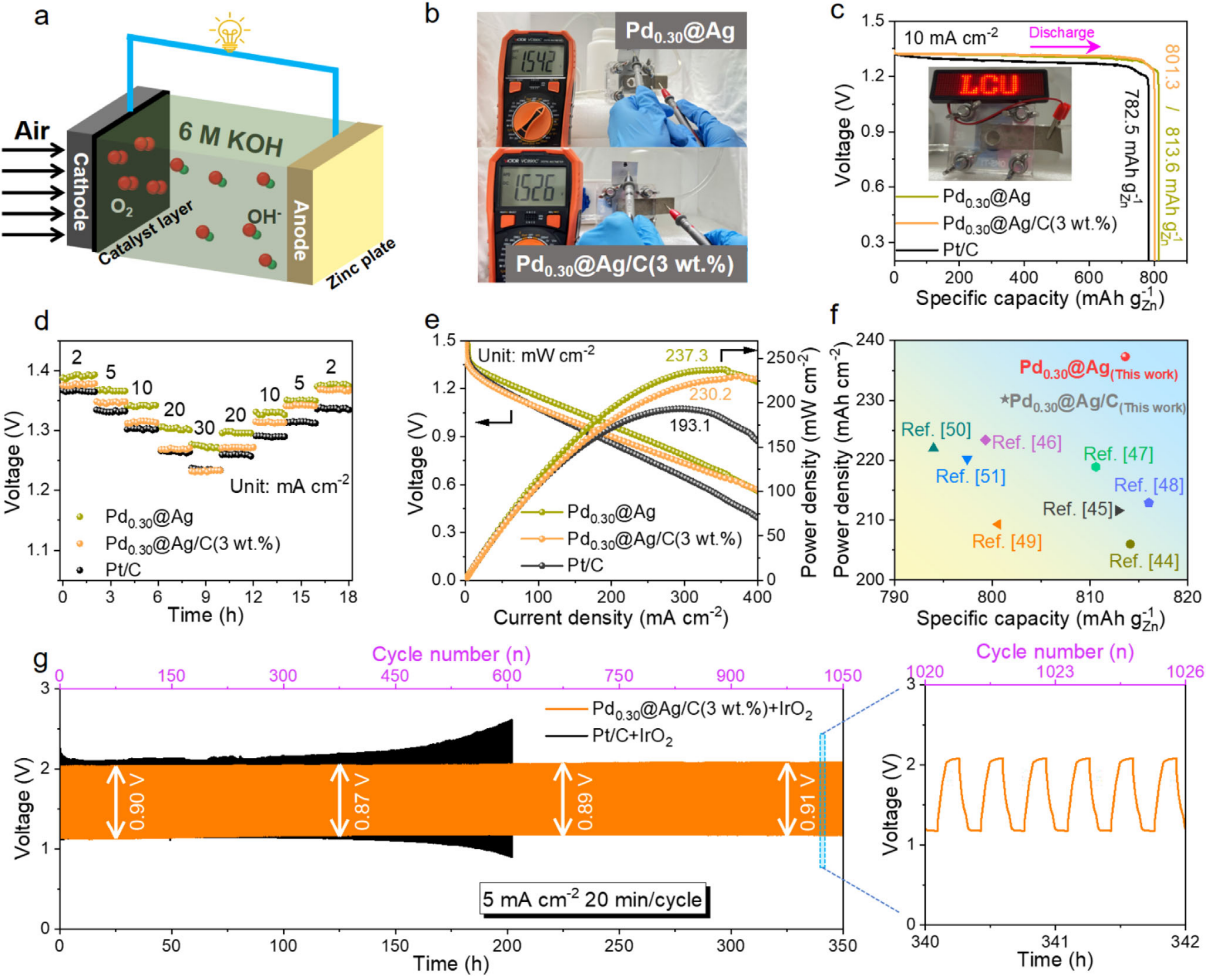
The ZABs measurement of electrocatalysts includes Pd_0.30_@Ag, Pd_0.30_@Ag/C(3 wt.%), and Pt/C. a) Schematic diagrams of home‐made ZAB. b) OCV measured with a multimeter. c) Specific capacity at a current density of 10.0 mA cm^−2^, inset photo of Pd_0.30_@Ag/C(3 wt.%)‐ZAB lights up an LED panel. d) Rate capability, and e) specific power versus current density of Pd_0.30_@Ag‐ZAB, Pd_0.30_@Ag/C(3 wt.%)‐ZAB, and Pt/C‐ZAB. f) Compare the power density and specific capacity of noble metal‐based catalytic materials reported in other studies with those presented in this work. g) Charge–discharge cycling performance of the Zn‐air battery, with a zoomed‐in view of voltage profiles.

Taking advantage of the excellent ORR performance of the electrocatalysts, zinc‐air batteries (ZABs) were assembled using carbon paper‐loaded electrocatalysts as the air cathode, 6.0 M KOH as the electrolyte, and a Zn plate as the anode (**Figure** **5**a). The open‐circuit voltage (OCV) of Pd_0.30_@Ag‐ZAB and Pd_0.30_@Ag/C(3 wt.%)‐ZAB were measured to be 1.542 and 1.526 V (Figure 5b), respectively, both higher than that of Pt/C‐ZAB (1.493 V) (Figure S14, Supporting Information), indicating superior catalytic activity.

The galvanostatic discharge curves at 10 mA cm^−2^ (Figure 5c) show that Pd_0.30_@Ag‐ZAB and Pd_0.30_@Ag/C(3 wt.%)‐ZAB exhibit specific capacities of 813.6 ${\mathrm{mAh}\;g}_{\mathrm{Zn}}^{-1}$ and 801.3 ${\mathrm{mAh}\;g}_{\mathrm{Zn}}^{-1}$, respectively, comparable to or exceeding that of Pt/C‐ZAB, highlighting their promise as Pt alternatives. The inset of Figure 5c displays a successful long‐term operation of an LED panel powered by a single Pd_0.30_@Ag/C(3 wt.%)‐ZAB. The rate capability of three ZABs was further tested under current densities ranging from 2 to 30 mA cm^−2^ (Figure 5d). Pd_0.30_@Ag‐ZAB maintained a higher voltage plateau across the entire current density range. Although Pd_0.30_@Ag/C(3 wt.%)‐ZAB showed a slight voltage drop at high current densities, its performance remained comparable to Pt/C‐ZAB. The maximum power densities achieved by Pd_0.30_@Ag‐ZAB and Pd_0.30_@Ag/C(3 wt.%)‐ZAB were 237.3 and 230.2 mW cm^−2^, respectively (Figure 5e), both significantly higher than that of Pt/C‐ZAB (193.1 mW cm^−2^). When compared with previously reported Pd‐based alloy catalysts, the Pd_0.30_@Ag‐based ZABs exhibit competitive power density and specific capacity, for practical and commercial applications (Figure 5f; Table S3, Supporting Information).^[^ ^44^, ^45^, ^46^, ^47^, ^48^, ^49^, ^50^, ^51^ ^]^ Even after undergoing over 1000 charge–discharge cycles under the current density of 5 mA cm^−2^, the ZAB cathode assembled by Pd_0.30_@Ag+IrO_2_ exhibited remarkable voltage stability with only a 1.11% voltage degradation (Figure 5g). In contrast, Pt/C+IrO_2_‐ZAB demonstrated significant voltage decay after ≈150 cycles, underscoring the superior durability of our catalyst. To evaluate the practical applicability under high‐current operating conditions, the charge–discharge behaviors of Pd_0.30_@Ag/C(3 wt.%)‐ZAB and Pt/C‐ZAB were also tested at 20 and 50 mA cm^−2^. Pd_0.30_@Ag/C(3 wt.%)‐ZAB demonstrates remarkable cyclic stability advantages over Pt/C‐ZAB at both current densities. Specifically, at 20 mA cm^−2^, Pd_0.30_@Ag/C(3 wt.%)‐ZAB maintains ≈90.2% of its initial discharge capacity after 300 cycles, significantly higher than Pt/C‐ZAB (≈65.3%) capacity retention (Figure S15a, Supporting Information). At 50 mA cm^−2^, Pd_0.30_@Ag/C(3 wt.%)‐ZAB shows negligible voltage degradation over the first 300 cycles, maintaining a stable discharge voltage profile (Figure S15b, Supporting Information). Whereas Pt/C‐ZAB exhibits abrupt voltage decay after ≈200 cycles. The coulombic efficiency remains consistently above 99% during charge–discharge cycles at different current densities (Figure S16, Supporting Information), indicating excellent reversibility and stability of the electrochemical reactions.

Achieving high round‐trip efficiency is crucial for the economic viability of rechargeable Zn–air batteries. Some hybrid systems have recently reported power densities exceeding 500 mW cm^−2^ under optimized alkali/acid conditions,^[^ ^52^ ^]^ demonstrating the field's progress. In contrast, our work focuses on a more practical and widely adopted alkaline configuration. This efficiency was evaluated based on charge–discharge voltage plateaus. As shown in Figure S17 (Supporting Information), the round‐trip efficiency of Pd_0.30_@Ag/C(3 wt.%)‐ZAB at the 300th cycle reached 57.6%, 49.2%, and 44.8% under current densities of 5, 20, and 50 mA cm^−2^, respectively. These values significantly surpass those of commercial Pt/C‐ZAB, which were 52.7%, 37.5%, and 24.9% under the same conditions. This indicates the excellent electrochemical reversibility and the long‐term durability of our catalyst system, highlighting the potential of Pd_0.30_@Ag/C as a cost‐effective and high‐performance alternative for practical energy storage applications.

To further evaluate the oxygen reduction performance under practical operating conditions, the Pd_0.30_@Ag/C (20 wt.%) and Pt/C (20 wt.%) catalysts were comparatively examined in anion‐exchange membrane fuel cells (AEMFCs). Membrane electrode assemblies (MEAs) with an active area of 4 cm^2^ and a total catalyst loading of 2.0 mg cm^−2^ were tested in single‐cell fixtures under H_2_/O_2_ and H_2_/air configurations at an absolute pressure of 0.2 MPa. Under H_2_/O_2_ operation, the Pd_0.30_@Ag/C‐based MEA delivered a peak power density of 496.9 mW cm^−2^, significantly surpassing the 441.3 mW cm^−2^ achieved by the Pt/C counterpart (Figure S18a, Supporting Information). Similarly, under more realistic H_2_/air conditions, the Pd_0.30_@Ag/C catalyst exhibited a remarkable peak power density of 362.8 mW cm^−2^, outperforming the Pt/C system (281.9 mW cm^−2^) by 28.7% (Figure S18b, Supporting Information). We further evaluated the performance retention of the catalyst after 30 000 cycles (Figure S19, Supporting Information). The peak power density retained 76.9% of its initial value (decrease of ∼115.3 mW cm^−2^) after 30 000 cycles, significantly outperforming commercially available Pt/C‐based H_2_‐O_2_ fuel cells, which showed a power density loss of ∼203.9 mW cm^−2^ (retention rate: 54.7%) under the same conditions. These compelling results robustly demonstrate the practical potential of Pd_0.30_@Ag as a promising alternative to Pt‐based metal catalysts in electrochemical catalysis.

## 3 Conclusion

In conclusion, we have successfully constructed interface‐confined quasi‐single Pd sites through oxygen‐driven surface reconstruction of PdAg alloy hollow nanotubes. Spectroscopic and theoretical analyses revealed that interfacial oxygen facilitates atomic rearrangement on the alloy surface, generating low‐coordinated Pd sites stabilized by oxygen, which serve as the key active centers for ORR. This structural modulation results in a negative shift of the *d*‐band center and effectively lowers the adsorption energy of ^*^OH intermediates, thereby promoting ORR kinetics. The Pd_0.30_@Ag catalyst delivers remarkable ORR activity and stability, comparable to or even surpassing commercial Pt/C at significantly lower Pd usage. Furthermore, the assembled zinc‐air batteries demonstrate high power density and excellent cycling durability. This study offers a valuable strategy to activate Pd‐based electrocatalysts via interface engineering and surface reconstruction, providing insights into the rational design of high‐efficiency ORR catalysts.

## 4 Experimental Section

### Materials and Methods

Polyvinylpyrrolidone (PVP‐K30, 98%), silver nitrate (AgNO_3_, 97%) and palladium (II) acetylacetonate (Pd(acac)_2_, 99%) were obtained from Shanghai Aladdin Biochemical Technology Co., Ltd. Sodium chloride (NaCl, 99%) was purchased from Tianjin Guangfu Fine Chemical Research Institute. Nafion solution (5 wt.%, Dupont) were bought from Sigma‐Aldrich Co., Ltd. Anhydrous ethanol (CH_3_CH_2_OH, 98%) and ammonia (NH_3_·H_2_O, 25–28 wt.%) were supplied by Yantai Yuandong Fine Chemicals Co., Ltd. Hydrochloric acid (HCl, 37 wt.%) was purchased from Tianjin Damao Chemical Reagent Factory. Ethylene glycol (EG, C_3_H_8_O, 99%) was bought from Beijing High Purity Technology Co., Ltd. Commercial platinum carbon (Pt/C, 20 wt.%) was purchased from Shanghai Hesen Electric Co., Ltd. All chemicals were used as received without further purification.

### Materials Characterizations

Powder X‐ray diffraction (XRD) analyses were performed with a Rigaku SmartLab 9 X‐ray diffractometer using Cu Kα radiation with λ = 1.5418 Å. Scanning electron microscopy (SEM) was conducted on a Thermo Fisher Scientific FIB‐SEM GX4. Transmission electron microscopy (TEM) was tested on JEOL2100. High‐angle annular dark‐field transmission electron microscopy (HAADF‐STEM) was operated on an FEI Titan Themis 80–200 (accelerating voltage: 200 kV) equipped with energy‐dispersive X‐ray spectroscopy (EDS). X‐ray photoelectron spectroscopy (XPS) was characterized using a Thermo Scientific ESCLAB 250Xi spectrometer with an Al‐Kα X‐ray source. Inductively coupled plasma optical emission spectrometry (ICP‐OES) measurement was performed on an Agilent Technologies 730 (OES) instrument. X‐ray absorption near‐edge spectroscopy (XANES) and extended X‐ray absorption fine structure (EXAFS): XANES and EXAFS experiments at 300 K were performed at the BL16U1 beam line at the Shanghai Synchrotron Radiation Facility (SSRF). Measurements of Pd/Ag k‐edges at ambient pressure were performed in high‐energy mode using gas ionization chambers to monitor incident and transmitted X‐ray intensity.

### Preparation Methods—*Preparation of Ag Nanowires (Ag NWs)*

PVP‐K30 (0.004 mmol, 200 mg) and AgNO_3_ (1 mmol, 182 mg) were dissolved in 15 mL of ethylene glycol (EG) and stirred vigorously for 30 min to form a homogeneous solution. Then NaCl (0.15 mmol, 9 mg) was added, and the mixture was stirred until complete dissolution of NaCl was achieved. The resulting solution was then transferred to a 20 mL autoclave and heated at 160 °C for 2 h. After natural cooling to room temperature, the obtained precipitate was collected and washed several times with anhydrous ethanol until the supernatant became clear, resulting in the formation of Ag nanowires (Ag NWs).

### Preparation Methods—*Preparation of Pd_x_@Ag (x = 0.15, 0.20, 0.25, 0.30)*

Ag NWs (0.4 mmol, 43 mg) were dispersed in 20 mL of a mixed solvent of ethanol and water with a volume ratio of 3:2. The pH of the dispersion was adjusted to 1 by the slow addition of 6 m HCl, followed by vigorous stirring for 1 h. Subsequently, the Pd(acac)_2_ solution configured in anhydrous ethanol (0.005 mm) was slowly injected into the Ag nanowire dispersion at a rate of 30 µL·h^−1^ using a microsyringe to initiate the galvanic displacement reaction. The product was then centrifuged, and 2 mL of NH_3_·H_2_O (10 wt.%) was added to remove the AgCl by‐product. The resulting alloy catalysts were thoroughly washed with deionized water and ethanol and then vacuum‐dried overnight to obtain Pd_x_@Ag. The extent of displacement reaction was adjusted by varying the amount of Pd(acac)_2_, where x denotes the molar ratio of Pd to Ag in the feedstock. The detailed material compositions for the synthesis of Pd_x_@Ag are summarized in Table S4 (Supporting Information).

### Preparation Methods—*Preparation of Pd_0.30_@Ag/C*

The as‐prepared Pd_0.30_@Ag hollow nanotubes were mechanically mixed with conductive carbon black (XC‐72) to fabricate carbon‐supported catalysts. Specifically, predetermined masses of Pd_0.30_@Ag (confirmed by ICP analysis) and XC‐72 were weighed to achieve target Pd mass fractions of 20%, 10%, 5%, and 3% in the final composites. The mixtures were manually ground in an agate mortar for 5 min to ensure homogeneous dispersion, followed by overnight drying at 60 °C under vacuum. The resulting composites were designated as Pd_0.30_@Ag/C(X, X = 20%, 10%, 5%, and 3 wt.%).

### Electrochemical Measurement

The electrochemical tests were carried out on the CHI 604E and CHI 760C workstations (Shanghai Chenhua Instrument Co., Ltd.). A standard three‐electrode system was employed with Ag/AgCl (3.0 m KCl) as the reference electrode, Pt wire as counter electrode, and a glassy carbon electrode as working electrode (disk area = 0.1256 cm^−2^, ring area = 0.1884 cm^−2^). All electrode potentials involved in the study were converted to the electrode potential of the standard reversible hydrogen electrode (RHE) according to the formula: $E_{\mathrm{RHE}}{=E}_{\mathrm{Ag}/\mathrm{AgCl}}^{\theta }+0.059\mathrm{pH}+E_{\mathrm{Ag}/\mathrm{AgCl}}$, without *iR* correction. The catalyst ink was prepared by ultrasonically dispersing the sample powder (2 mg) in a solvent mixture (V_ethanol_:V_water_ = 1:1) and then adding 10 µL Nafion (5 wt.%). This ink was coated onto the polished glassy carbon electrode with a Pd loading of 0.03 mg_Pd_ cm^−2^. During the accelerated durability test (ADT), cyclic voltammetry (CV) curves were performed in the voltage range of 0.66 to 1.06 V versus RHE at a scan rate of 100 mV s^−1^ for 5000 cycles. The electrocatalytic stability was evaluated by comparing the changes in linear sweep voltammetry (LSV) polarization curves at 1600 rpm before and after ADT.

The electron transfer number (n) is calculated based on the LSV at different scan rates according to the K‐L equation:

(7) $$\frac{1}{J}=\frac{1}{J_{K}}+\frac{1}{J_{L}}=\frac{1}{J_{K}}+\frac{1}{B\omega^{1/2}}$$

(8) $$J_{K}={\mathrm{nFkC}}_{O_{2}}^{*}\;$$

(9) $$B\;=0{.62\mathrm{nFC}}_{O_{2}}^{*}D_{O_{2}}^{2/3}v^{-1/6}$$

where J represents the measured current density, J_L_ and J_K_ denote the diffusion‐limited current density and kinetic current density, respectively. In addition, F, ω, and v are the Faraday constant (F = 96485C mol^−1^), the electrode rotation rate, and the kinematic viscosity of the electrolyte (0.01 cm^2^ s^−1^), respectively. The bulk concentration is denoted as $C_{O_{2}}^{*}$ (1.2 × 10^−6^ mol cm^−3^), while the diffusion coefficient is given by $D_{O_{2}}$ (1.9 × 10^−5^ cm^2^ s^−1^).

The n and H_2_O_2_ yield can also be determined by the rotating ring disk electrode (RRDE) equations as follows:

(10) $$n=\frac{4I_{D}}{I_{D}+I_{N}/N}\;$$

(11) $$H_{2}O_{2}\;\left(\%\right)=200\times \frac{I_{R}/N}{I_{D}+I_{R}/N}$$

where I_R_ and I_D_ represent the ring current and disk current, respectively. N is the current collection efficiency of the Pt ring (0.44).

### Theoretical Calculation Methods and Models

The geometry structures were optimized using Density Functional Theory (DFT) as implemented in the Vienna Ab‐initio Simulation Package (VASP). All geometry optimizations and self‐consistent total energy calculations were conducted with the projector‐augmented wave (PAW) method based on the Perdew‐Burke‐Ernzerhof (PBE) density functional. An energy convergence criterion of 10^−6^ eV was employed for self‐consistent calculations with the kinetic energy cut‐off set at 500 eV, while atomic positions were relaxed until the Hellmann‐Feynman forces converged to 0.01 eV Å^−1^. The first Brillouin zone was sampled using a 3 × 3 × 1 Monkhorst‐Pack k‐point mesh to ensure accurate calculations of the electronic properties. The vacuum layer was set to 20 Å, which was enough to prevent interactions between periodically repeated structures in the z‐direction. The model employed a supercell structure with a 4 × 4 expansion, fixing the atoms of the bottom layer while relaxing the two layers closest to the active interface.

### Assembly and Testing of Zn–Air Batteries (ZABs)

The ZABs were assembled using a 0.2 mm thick zinc plate as the anode, 6.0 m KOH as the electrolyte, and Pd_x_@Ag or commercial Pt/C uniformly coated on carbon paper as the air cathode (effective area: 1 cm^−2^). The catalyst ink was prepared by dispersing 2.0 mg of catalyst in a mixed solution of 200 µL ethanol and 5 µL Nafion (5 wt.%). The resulting ink was uniformly coated onto the carbon paper with a catalyst loading of 1 mg cm^−1^. The power density curves were measured using CHI 760C, while the galvanostatic discharge data at a current density of 10 mA cm^−2^ were recorded with the LANHE‐CT2001A battery testing system. Additionally, discharge performance at different current densities of 2, 5, 10, 20, and 30 mA cm^−2^ was evaluated to assess the rate capability of the batteries.

### Optimized Fabrication of Membrane Electrode Assembly and Fuel Cell Testing

The electrochemical performance of Pd_0.30_@Ag/C‐based anion‐exchange membrane fuel cells (AEMFCs) was systematically evaluated using a Darkstream AL‐CS‐150 test platform. The catalyst‐coated membrane (CCM) fabrication involved sequential deposition of electrode layers through the following procedure: 1) Cathode preparation: A homogeneous ink containing Pd_0.30_@Ag/C catalyst (20 wt.%, Pd loading 1.0 mg cm^−2^) and ionomer binder (4:1 mass ratio) was dispersed in *n*‐propanol; 2) Anode preparation: A Pt/C‐based ink (20 wt.%, Pt loading 1.0 mg cm^−2^) with identical binder ratio and solvent system was formulated. Both electrode inks were uniformly deposited on respective sides of the preheated membrane (60 °C substrate temperature) through precision spray‐coating, achieving an active area of 4 cm^2^ (2 × 2 cm^2^).

Prior to cell assembly, the CCM underwent critical ion‐exchange treatment through immersion in 6 m KOH solution at 60 °C for 4 h, followed by thorough deionized water rinsing to ensure complete Cl^−^/OH^−^ replacement. The membrane‐electrode assembly (MEA) was completed by integrating two Toray carbon paper gas diffusion layers (GDLs, 280 µm thickness) with the conditioned CCM, employing bipolar plates and PTFE gaskets under controlled torque application (5 N·m). The Pt/C‐MEA used as the cathode in H_2_–Air fuel cells follows the same preparation protocol as the anode in H_2_–O_2_ cells, ensuring consistency in experimental conditions.

Fuel cell evaluation was conducted under optimized operating conditions: 80 °C cell temperature, 100% relative humidity for both reactant gases (H_2_/O_2_, flow rate of 500 standard cubic centimeters per minute), and balanced backpressure regulation (0.2 MPa for both electrodes). Current‐voltage polarization curves were acquired after 2 h activation at 0.5 V to ensure stable operation.

### Statistical Analysis

The “Statistical Analysis” section has been added to the manuscript: No statistical hypothesis testing or post‐hoc analysis was performed. Electrochemical and battery measurements were repeated independently at least three times (*n* ≥ 3) to ensure reproducibility. Representative results are presented in the manuscript. No data pre‐processing (e.g., transformation, normalization, or outlier exclusion) was applied. Quantitative values are reported without error bars. Data processing and visualization were performed using OriginPro 2022.

## Conflict of Interest

The authors declare no conflict of interest.

## Supporting information



Supporting Information

## Data Availability

The data that support the findings of this study are available from the corresponding author upon reasonable request.
