# Supplementary material for: Oxygen‐Driven Reconstruction Activates Quasi‐Single Pd Sites in Hollow PdAg Nanotubes for Zinc–Air and Fuel Cells
Source: Adv Sci (Weinh). 2025 Jul 17;12(43):e09329. doi: 10.1002/advs.202509329 (PMC12631890; doi:10.1002/advs.202509329)
Supplement: Supplementary file 1 — Supporting Information [file ADVS-12-e09329-s001.docx]

Supporting Information

**Oxygen-Driven Reconstruction Activates Quasi-Single Pd Sites in Hollow PdAg Nanotubes for Zinc–Air and Fuel Cells**

Zongge Li, Wenjie Tian, Kunsheng Hu, Yajie Guo, Xiaotan Tian, Wenjun Kang, Rui Li, Konggang Qu, Lei Wang, Fanpeng Meng,* Huayang Zhang,* Haibo Li*

Dr. Z. Li, Y. Guo, X. Tian, R. Li, Y. Yang, W. Kang, R. Li, K. Qu, L. Wang, F.Meng, Prof. H. Li
Shandong Provincial Key Laboratory of Chemical Energy Storage and Novel Cell Technology,
School of Chemistry and Chemical Engineering, Liaocheng University,
Liaocheng 252059, Shandong, China
E-mail: [mengfanpeng@lcu.edu.cn](mailto:mengfanpeng@lcu.edu.cn) (F. Meng) and [haiboli@mail.ustc.edu.cn](mailto:haiboli@mail.ustc.edu.cn) (H. Li)

Dr. W. Tian, K. Hu, H. Zhang
School of Chemical Engineering, The University of Adelaide,
Adelaide SA 5005, Australia
E-mail: [huayang.zhang@adelaide.edu.au](mailto:huayang.zhang@adelaide.edu.au) (H. Zhang)

**Table of Contents**

[**1. Catalysts preparation 3**](#_Toc201225486)

[**2. Electron microscopic characterisations 4**](#_Toc201225487)

[**3. Characterisations of structure and composition 8**](#_Toc201225488)

[**4. Electrochemical tests 10**](#_Toc201225489)

[**5. Information on the structure of models calculated by DFT 14**](#_Toc201225490)

[**6. Test and Evaluation of Fuel Cell Performance 16**](#_Toc201225491)

[**7. The pertinent parameters in the tables 22**](#_Toc201225492)

# 1. Catalysts preparation


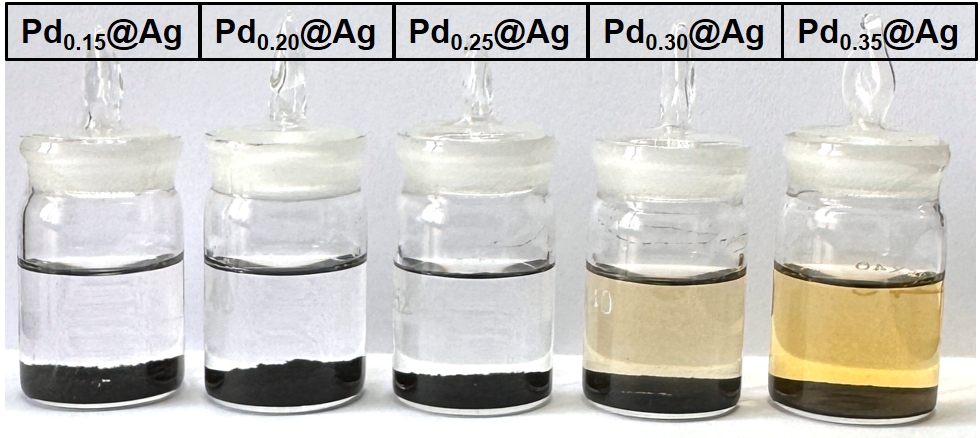


**Figure S1.** Color change of the solution with increasing molar concentration of Pd^2+^.

# 2. Electron microscopic characterisations


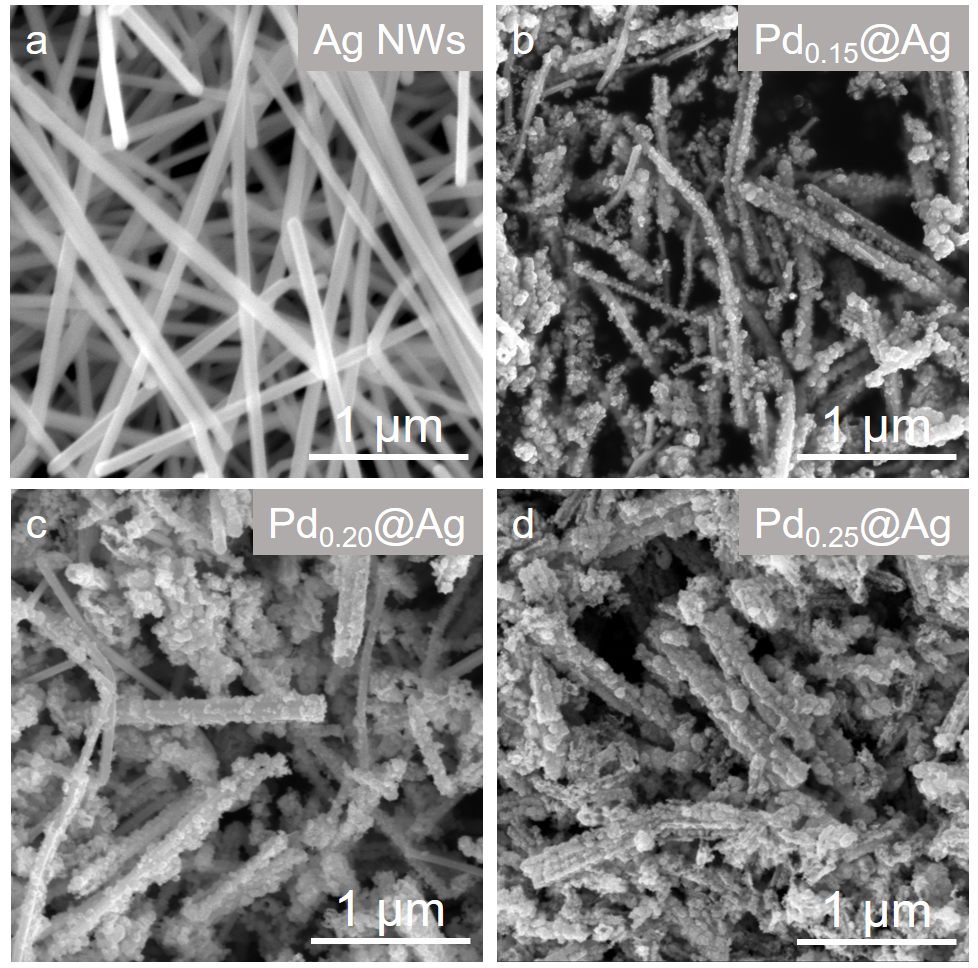


**Figure S2.** SEM images of the as-prepared (a) Ag NWs and Pd_x_@Ag with varying Pd loadings, specifically (b) Pd_0.15_@Ag, (c) Pd_0.20_@Ag and (d) Pd_0.25_@Ag.


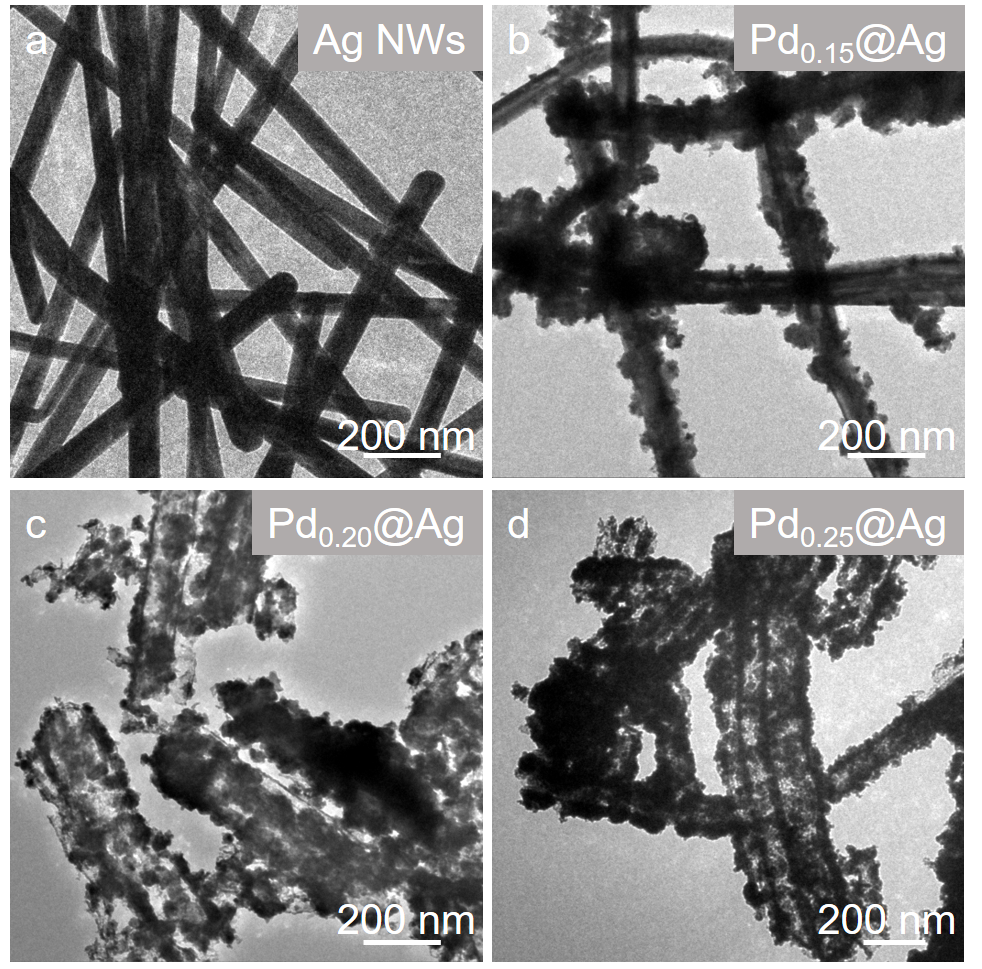


**Figure S3.** TEM images of the as-prepared (a) Ag NWs, (b) Pd_0.15_@Ag, (c) Pd_0.20_@Ag, and (d) Pd_0.25_@Ag.


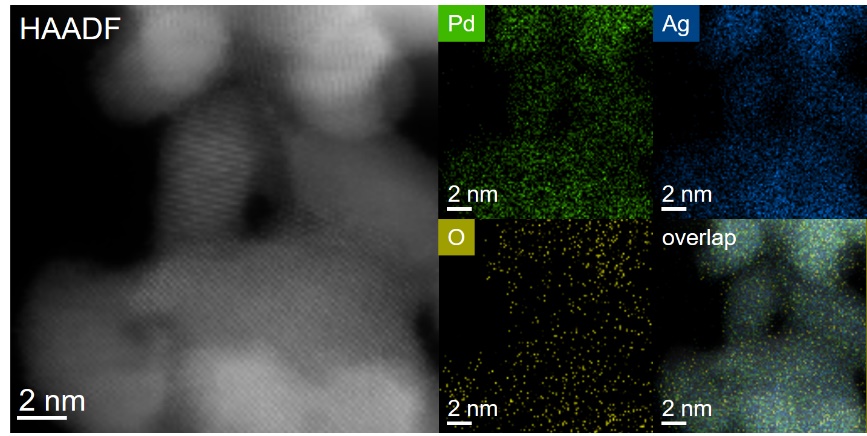


**Figure S4.** AC-HAADF-STEM image and corresponding EDS mapping images of the elements Ag, Pd and O.


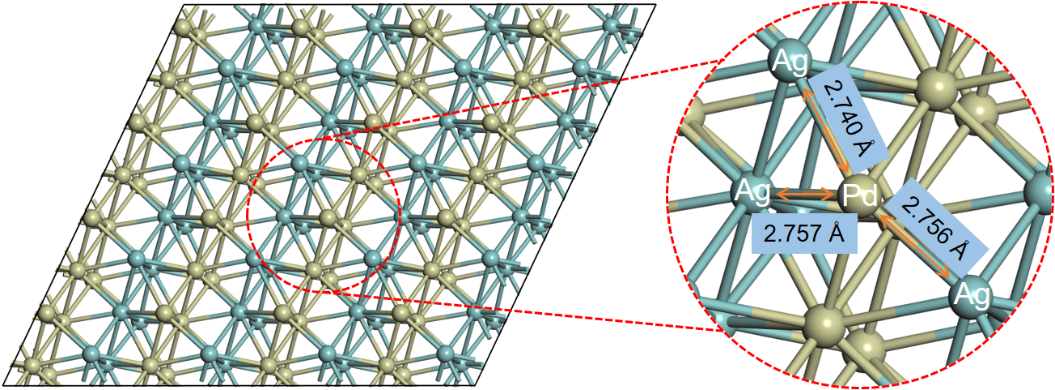


**Figure S5.** Bond length analysis of DFT-optimized PdAg alloy models on the (111) crystal planes.

# 3. Characterisations of structure and composition

**Figure S6.** XPS survey spectra and ICP of Ag NWs, Pd_0.15_@Ag, Pd_0.20_@Ag, Pd_0.25_@Ag and Pd_0.30_@Ag.

**Figure S7.** Wavelet transform (WT) of Pd foil, PdO, Ag foil and Ag_2_O.

# 4. Electrochemical tests

**Figure S8.** CV curves at 20 mV s^-1^ of Ag NWs, Pd_0.15_@Ag, Pd_0.20_@Ag, Pd_0.25_@Ag, Pd_0.30_@Ag and Pt/C.

**Figure S9.** Equivalent circuit diagrams for EIS fitting.

**Figure S10.** LSV curves of (a) Ag NWs, (b) Pd_0.15_@Ag, (c) Pd_0.20_@Ag, (d) Pd_0.25_@Ag, (e) Pd_0.30_@Ag and (f) Pt/C at different rotation speeds. Corresponding K-L plots of (g) Ag NWs, (h) Pd_0.15_@Ag, (i) Pd_0.20_@Ag, (j) Pd_0.25_@Ag, (k) Pd_0.30_@Ag and (l) Pt/C.

**Figure S11.** LSV curves measured at RRDE.

# 5. Information on the structure of models calculated by DFT


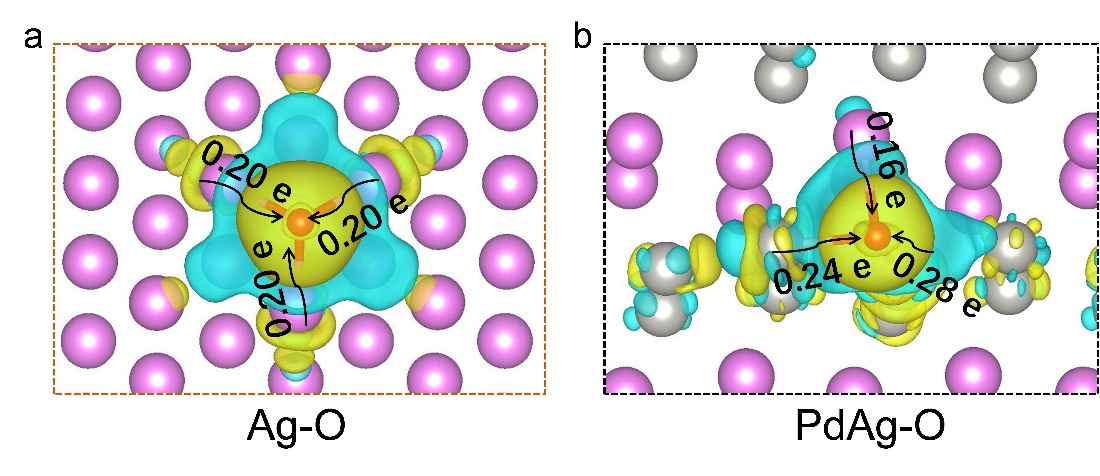


**Figure S12.** The electron shift induced by O adsorption (yellow represents electron enrichment region, while blue indicates electron-depleted region).


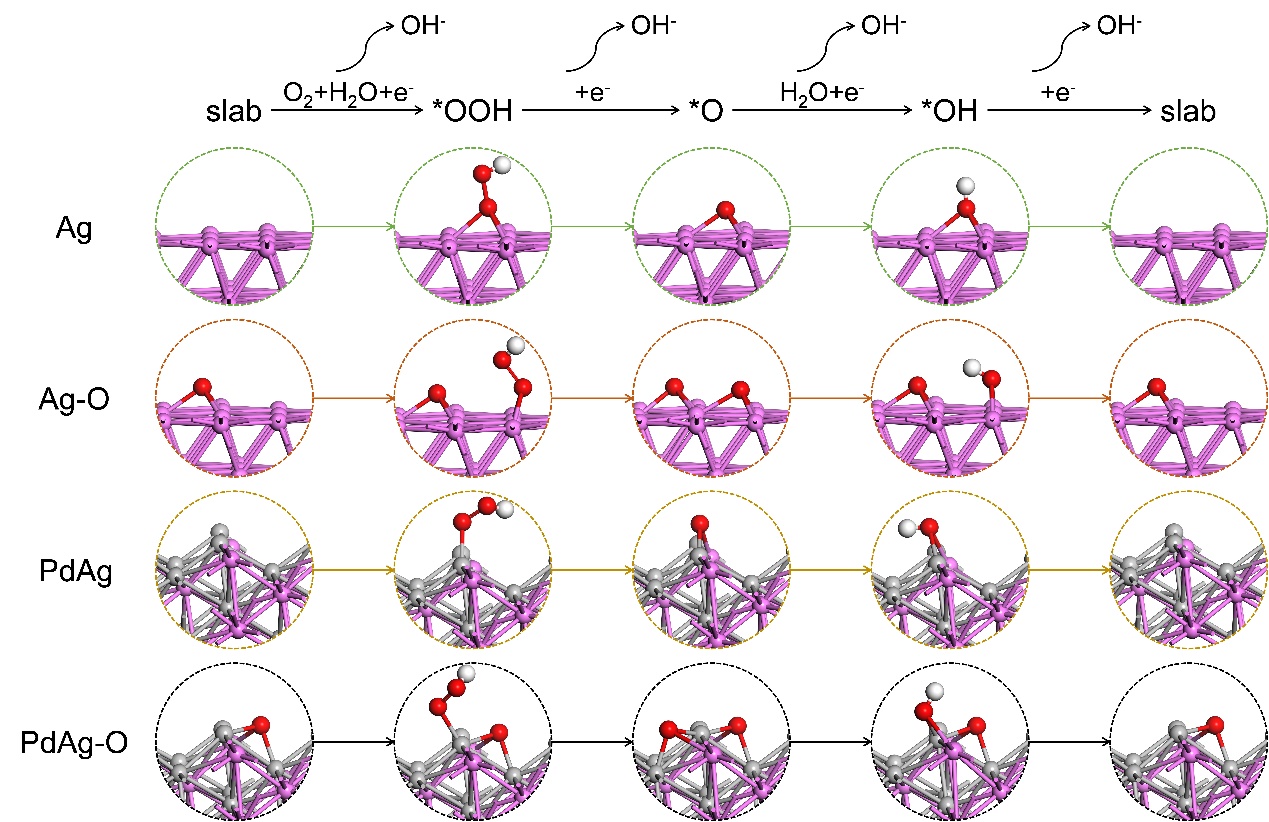


**Figure S13.** Schematic diagrams of the ORR pathways of Ag (111), Ag-O (111), PdAg (111) and PdAg-O (111).

# 6. Test and Evaluation of Fuel Cell Performance

**Figure S14.** The OCV of Pt/C-ZAB

**Figure S15.** Charge-discharge cycles for Pd_0.30_@Ag/C-ZAB and Pt/C-ZAB at current densities of (a) 20 mA cm^-2^ and (b) 50 mA cm^-2^.

**Figure S16.** Coulombic efficiency for Pd_0.30_@Ag/C and Pt/C during charge-discharge cycles at current densities of 5 mA cm^-2^, 20 mA cm^-2^, and 50 mA cm^-2^.

**Figure S17.** Voltage round-trip efficiency of Pd_0.30_@Ag/C and Pt/C at the 300th cycle under different current densities.

**Figure S18.** (a) H_2_-O_2_ and (b) H_2_-air fuel cells performance measured at 80°C and 100% R.H. under 180 kPa abs.

**Figure S19.** The power density of H_2_-O_2_ fuel cells performance for Pd_0.30_@Ag/C and Pt/C catalysts before and after 30,000 cycles.

# 7. The pertinent parameters in the tables

**Table S1.** EXAFS fitting parameters at the Pd and Ag K-edge for various samples.

| Sample | | Shell | CN^a^ | R(Å)^b^ | σ^2^(Å^2^)^c^ | ∆E_0_(eV)^d^ | R factor |
| --- | --- | --- | --- | --- | --- | --- | --- |
| Pd K-edge (S_0_^2^=0.820) | | | | | | | |
| Pd foil | | Pd-Pd | 12* | 2.737±0.001 | 0.0054±0.0003 | -0.1±0.5 | 0.0031 |
| PdO | | Pd-O | 6.0±0.1 | 2.149±0.025 | 0.0027±0.0032 | -18.6±4.5 | 0.0236 |
|  |  | Pd-Pd | 9.4±0.3 | 3.226±0.016 | 0.0007±0.0026 | -0.7±2.6 |  |
| Pd_0.30_@Ag | | Pd-O | 1.1±0.2 | 1.997±0.008 | 0.0012±0.0011 | 3.2±1.4 | 0.0224 |
|  |  | Pd-Pd | 7.1±0.4 | 2.724±0.006 | 0.0033±0.0007 | -2.7±0.6 |  |
|  |  | Pd-Ag | 4.1±0.3 | 2.796±0.005 | 0.0016±0.0002 | -21.1±3.9 |  |
| Ag K-edge (S_0_^2^=0.632) | | | | | | | |
| Ag foil | Ag-Ag | | 12* | 2.861±0.002 | 0.0099±0.0005 | 0.5±0.2 | 0.0071 |
| Ag_2_O | Ag-O | | 3.9±0.2 | 2.216±0.044 | 0.0125±0.0071 | -6.0±4.8 | 0.0046 |
|  | Ag-Ag | | 9.8±0.4 | 3.438±0.051 | 0.0171±0.0072 | -4.2±3.4 |  |
| Pd_0.30_@Ag | Ag-O | | 1.0±0.1 | 2.013±0.006 | 0.0122±0.0016 | -16.7±4.9 | 0.0135 |
|  | Ag-Pd | | 6.5±0.3 | 2.851±0.008 | 0.0149±0.0058 | -6.3±2.7 |  |
|  | Ag-Ag | | 5.1±0.3 | 2.826±0.005 | 0.0074±0.0013 | -10.9±5.3 |  |

*^a^CN*, coordination number; *^b^R*, the distance to the neighboring atom; *^c^σ*^2^, the Mean Square Relative Displacement (MSRD); *^d^ΔE*_0_, inner potential correction; *R* factor indicates the goodness of the fit.

**Table S2** The simulated equivalent circuit data of the catalysts

| **Catalysts** | **R_s_ (Ω cm^-2^)** | **R_ct_ (Ω cm^-2^)** | **CPE-T** | **CPE-P** |
| --- | --- | --- | --- | --- |
| Pd_0.30_@Ag | 5.913 | 49.22 | 9.895×10^-9^ | 1.01 |
| Pd_0.25_@Ag | 10.87 | 62.38 | 2.58×10^-9^ | 1.031 |
| Pd_0.20_@Ag | 9.146 | 69.51 | 3.31×10^-9^ | 1.018 |
| Pd_0.15_@Ag | 8.408 | 90.75 | 2.919×10^-9^ | 1.008 |
| Ag NWs | 10.72 | 109.8 | 2.23×10^-9^ | 1.01 |

**Table S3** Comparison of performance for Pd-based catalyst-assembled Zn-air batteries

| **Catalysts** | **Open-circuit voltage (V)** | **Power density**  **(mW cm^-2^)** | **Specific capacity**  **(mAh** $\mathbf{g}_{\mathbf{Zn}}^{\mathbf{-1}}$**)** | **Ref.** |
| --- | --- | --- | --- | --- |
| Pd_0.30_@Ag | 1.542 | 237.3 | 813.6 | This work |
| Pd_0.30_@Ag/C(3 wt.%) | 1.526 | 230.2 | 801.3 | This work |
| Pd_45_Pt_44_Ni_11_SpNSs/C | 1.49 | 206 | 814.1 | [1] |
| PdNiMnO | 1.37 | 211.6 | 812.9 | [2] |
| Pd_25%_Te_75%_ | 1.535 | 223.4 | 799.3 | [3] |
| Pd_25%_Sn_75%_-NPC | 1.514 | 218.9 | 810.6 | [4] |
| o-Pd_3_Fe@NC | 1.46 | 212.9 | 816 | [5] |
| Bi−Pd/C metallene | 1.546 | 209.3 | 800.3 | [6] |
| *o*-PdTe@Pd | 1.442 | 222 | 794 | [7] |
| Pd NM/C | 1.440 | 220.23 | 797.43 | [8] |

**Table S4** The amount of raw materials used for the preparation of Pd_x_@Ag

| Raw materials  Catalysts | Ag NWs | Pd(acac)_2_ |
| --- | --- | --- |
| Pd_0.15_@Ag | 0.4 mmol, 43mg | 0.06 mmol, 18.30 mg |
| Pd_0.20_@Ag |  | 0.08 mmol, 24.40 mg |
| Pd_0.25_@Ag |  | 0.10 mmol, 30.50 mg |
| Pd_0.30_@Ag |  | 0.12 mmol, 36.63 mg |

**References**

[1] K. Liu, H. Huang, Y. Zhu, S. Wang, Z. Lyu, X. Han, Q. Kuang, S. Xie, *J. Mater. Chem. A* **2022**, *10*, 3808-3817.

[2] W. Zhang, J. Chang, G. Wang, Z. Li, M. Wang, Y. Zhu, B. Li, H. Zhou, G. Wang, M. Gu, Z. Feng, Y. Yang, *Energy Environ. Sci.* **2022**, *15*, 1573-1584.

[3] Y. Guo, F. Zheng, T. Wang, X. Liu, X. Tian, K. Qu, L. Wang, R. Li, W. Kang, Z. Li, H. Li, *ACS Appl. Mater. Interfaces* **2024**, *16*, 36363-36372.

[4] Z. Li, J. Chen, Y. Guo, F. Zheng, K. Qu, L. Wang, R. Li, S. Xiong, W. Kang, H. Li, *J. Colloid Interface Sci.* **2024**, *659*, 257-266.

[5] S. Ghora, B. Satpati, B. K. Jena, C. R. Raj, *ACS Appl. Mater. Interfaces* **2025**, *17*, 3426-3435.

[6] L. Xie, J. Wang, K. Wang, Z. He, J. Liang, Z. Lin, T. Wang, R. Cao, F. Yang, Z. Cai, Y. Huang, Q. Li, *Angew. Chem., Int. Ed.* **2024**, *63*, e202407658.

[7] M. Song, Q. Zhang, T. Shen, G. Luo, D. Wang, *Chin. Chem. Lett.* **2024**, *35*, 109083.

[8] J. Tian, Y. Song, X. Hao, X. Wang, Y. Shen, P. Liu, Z. Wei, T. Liao, L. Jiang, J. Guo, B. Xu, Z. Sun, *Adv. Mater.* **2025**, *37*, 2412051.
